# A Wider Pelvis Does Not Increase Locomotor Cost in Humans, with Implications for the Evolution of Childbirth

**DOI:** 10.1371/journal.pone.0118903

**Published:** 2015-03-11

**Authors:** Anna G. Warrener, Kristi L. Lewton, Herman Pontzer, Daniel E. Lieberman

**Affiliations:** 1 Department of Human Evolutionary Biology, Harvard University, 11 Divinity Avenue, Cambridge, Massachusetts, 02138, United States of America; 2 Department of Anatomy and Neurobiology, Boston University School of Medicine, 72 East Concord St. (L 1004), Boston, Massachusetts, 02118, United States of America; 3 Department of Anthropology, Hunter College, 695 Park Ave., New York, New York, 10065, United States of America; University of Utah, UNITED STATES

## Abstract

The shape of the human female pelvis is thought to reflect an evolutionary trade-off between two competing demands: a pelvis wide enough to permit the birth of large-brained infants, and narrow enough for efficient bipedal locomotion. This trade-off, known as the obstetrical dilemma, is invoked to explain the relative difficulty of human childbirth and differences in locomotor performance between men and women. The basis for the obstetrical dilemma is a standard static biomechanical model that predicts wider pelves in females increase the metabolic cost of locomotion by decreasing the effective mechanical advantage of the hip abductor muscles for pelvic stabilization during the single-leg support phase of walking and running, requiring these muscles to produce more force. Here we experimentally test this model against a more accurate dynamic model of hip abductor mechanics in men and women. The results show that pelvic width does not predict hip abductor mechanics or locomotor cost in either women or men, and that women and men are equally efficient at both walking and running. Since a wider birth canal does not increase a woman’s locomotor cost, and because selection for successful birthing must be strong, other factors affecting maternal pelvic and fetal size should be investigated in order to help explain the prevalence of birth complications caused by a neonate too large to fit through the birth canal.

## Introduction

The human pelvis is a complex structure whose form reflects the demands of locomotion, climatic adaptation [1,2], support of the viscera [3], and in females, birth. Because of these multiple influencing factors, the pelvis is often thought to be under competing selective demands requiring functional trade-offs. Perhaps most significantly, bipedal locomotion and human childbirth have long been argued to have especially strong antagonistic effects on the female pelvis [4–11]. A narrow pelvis is thought to increase locomotor efficiency [4–6,10,12] while a wide pelvis increases the capacity of the birth canal, reducing the risk of obstructed labor. Maintaining a spacious birth canal was likely particularly important by the Middle Pleistocene when brain size began to increase rapidly in the genus *Homo*[13,14] affecting neonatal cranial size [15]. However, minimizing locomotor cost may also be particularly important to female reproductive fitness. Women in hunter-gatherer societies are known to regularly travel 5 km or more a day, often carrying substantial loads[16,17], and they also must maintain adequate energetic resources for pregnancy and lactation. These conflicting evolutionary demands on the female pelvis are thought to be at least partially responsible for the difficulty of modern human childbirth and the occurrence of cephalopelvic disproportion [4,7–9]. This trade-off scenario, often referred to as the obstetrical dilemma, has important consequences for understanding the human birth process, maternal investment and infant development [5,18]. Despite the wide acceptance of the obstetrical dilemma model, the effect of increased pelvic width on locomotor cost has never been directly addressed. Here we present experimental data that calls into question the standard static biomechanical model relating pelvic width to locomotor cost and present a more accurate model of the effect of dynamic hip mechanics on the cost of walking and running.

A unique biomechanical challenge of humans’ striding bipedal gait is balancing the body over a single supporting limb during walking and running. Because the hip joint lies some distance from the body’s midline, the pelvis tends to rotate away from the supporting side during single-leg support. The hip abductor muscles (gluteus medius, gluteus minimus and tensor fasciae latae) counteract this rotation by producing an opposing force on the pelvis thereby redirecting the body center of mass to maintain mediolateral balance. In order to maintain equilibrium about the hip joint, the external moment acting about the hip in the mediolateral plane must be opposed by an equal and opposite internal moment, generated primarily by the hip abductor muscles.

**Fig 1:**
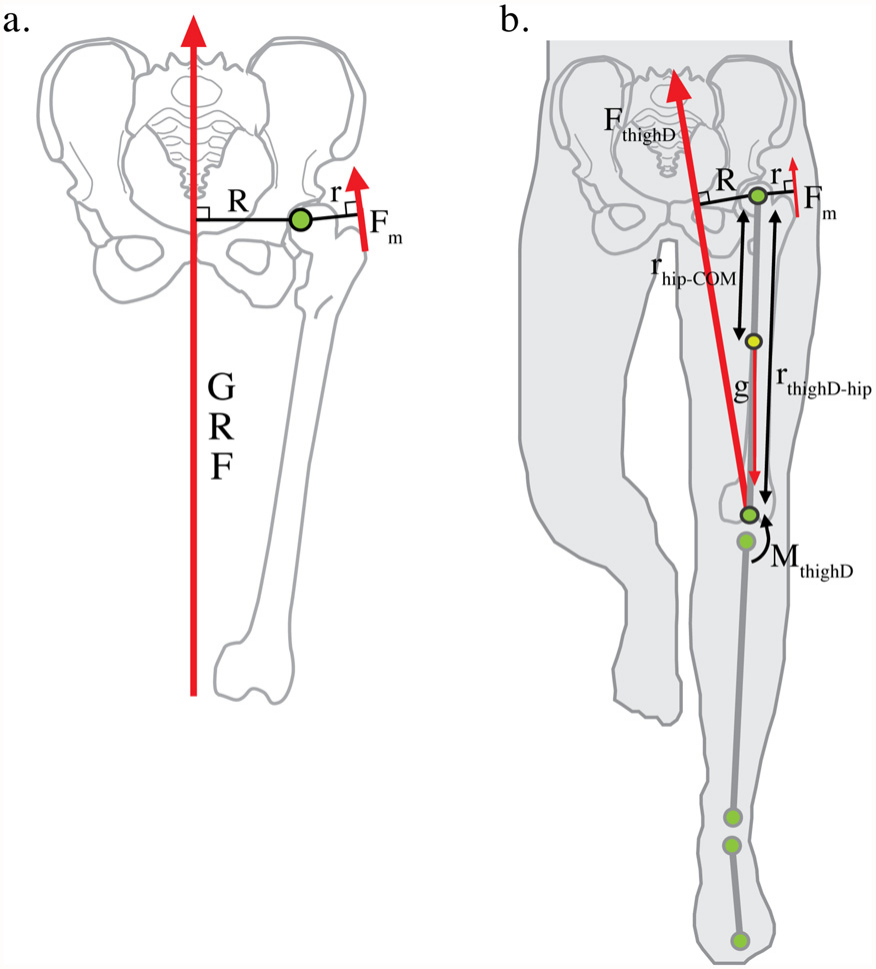
Static and dynamic models of hip abductor force production. a. The standard static biomechanical model of hip abductor force production assumes the ground reaction force vector (*GRF*) passes nearly vertically through the body center of mass during locomotion. The effective mechanical advantage (*EMA*) of the hip abductor muscles is defined as *r*/*R*, where *r* is the abductor muscle moment arm and *R* is the moment arm of the *GRF* vector. Hip abductor force (*F*_*m*_) is equal the multiple of *GRF* and 1/*EMA*. A narrow pelvis is thought to reduce locomotor cost by decreasing *R* and hip abductor force production, but this may increase the likelihood of cephalopelvic disproportion (CPD) by narrowing the maternal birth canal. b. Inverse dynamics models the lower extremity as a series of linked-segments in which the foot, shank and thigh each act as rigid bodies interacting at frictionless joints [23,24]. The net internal hip moment is equal to the thigh moment of inertia times its angular acceleration, minus the distal thigh moment (*M*_*thighD*_), minus the cross-product of the positional vector of hip relative to the thigh center of mass (*r*_*hip-COM*_) and gravity (*g*), minus the external moment acting on the thigh defined as the cross product of the positional vector of the distal thigh segment relative to the hip (*r*_*thighD-hip*_) and the force vector acting on the distal thigh (*F*_*thighD*_). *R* can then be measured dynamically as the perpendicular distance from the hip joint center to *F*_*thighD*_ (see Methods). If the predictions of the static model and the obstetrical dilemma hold, *R* measured dynamically will be nearly equal to half- biacetabular width.

Under the standard static biomechanical model [12,19–22], abductor muscle force, *F*_*m*_, is determined by the magnitude of the external force, the ground reaction force (*GRF*), and the effective mechanical advantage (*EMA*) of the hip abductors: the ratio of the hip abductor muscle moment arm, *r*, to the *GRF* moment arm, *R* (Fig. 1a). Because this model assumes that the *GRF* passes nearly vertically through the body center of mass at mid-stance of gait, *R* is thought to be approximately equal to half biacetabular width [12,19]. Biacetabular width is traditionally measured from the innermost aspect of the acetabulum. However, since hip joint rotation occurs about the center of the femoral head, we define biacetabular width as the distance between the centers of the femoral heads, a more biomechanically relevant measure for understanding hip abductor function[12,19]. If half biacetabular width is a good proxy for *R* during locomotion as the standard model assumes, then increasing this pelvic diameter will decrease abductor *EMA* and, assuming no change in *GRF* and *r*, require more muscle force and greater metabolic energy to maintain pelvic stability during the single leg support phase of walking and running [12,19,20]. Therefore, the obstetrical dilemma predicts that greater pelvic width in females associated with the demands of birthing large brained infants compromises hip abductor *EMA* and results in less efficient locomotion in women compared to men [4,5,8,12].

Inverse dynamics provides an alternative approach for estimating dynamic hip abductor force production during walking and running by integrating *GRF* data from a force platform, which provides information on whole body center of mass accelerations, with kinematic data from each segment of the lower extremity. Briefly, the method models the lower extremity as a series of linked-segments in which the foot, shank and thigh each act as rigid bodies interacting at frictionless joints [23,24]. Because the external force acting on the limb model (the *GRF*) and the kinematics of each segment are known, the moments about each joint responsible for the observed angular accelerations of the segments can be resolved using Newton-Euler equations [23] (Fig. 1b; see Methods). Beginning with the distal-most segment (the foot), the center of mass accelerations of each segment are subtracted from the whole so that calculations at the hip represent the acceleration of the whole body minus the accelerations of each segment of the stance limb. This approach permits dynamic measurements of the moment about the hip, *R*, due to the external forces acting on the thigh in the coronal plane, and hip abductor force production during locomotion. Previous studies have failed to establish consistent differences in walking and running economy between women and men [25–27], but because the effect of greater biacetabular width on dynamic hip abductor mechanics and locomotor energetics are poorly established [18], one of the core tenets of the obstetrical dilemma remains untested.

## Materials and Methods

Use of human subjects was approved by IRB review of the Human Research Protection Programs at both Harvard University (#17229) and Washington University in St. Louis (#09–0216), and all subjects provided written consent prior to participation in the study. Subjects were grouped by sex based on their self-identification at the time of initial testing.

Two experiments were conducted, one at Harvard University and one at Washington University in St. Louis. In the first experiment, metabolic data was collected on fifteen subjects (male n = 8, female n = 7) in order to compare net locomotor cost between men and women. In the second experiment, we collected kinematic, kinetic, and magnetic resonance imaging (MRI) data in addition to metabolic data on twenty-six individuals (male n = 13, female n = 13) in order to examine how pelvic width and hip abductor *EMA* affects locomotor cost. Subjects were all physically fit recreational runners and non-smokers between 20–35 years of age.

### Kinematics and kinetics

Subjects walked and ran over an AMTI model-OR force-platform (1000Hz) embedded halfway down a 7.8m long track-way while kinematics (200Hz, Vicon) data were simultaneously recorded. Data from trials in which only a single foot made contact with the force-platform, speed was constant (defined as the difference between absolute horizontal breaking and accelerating impulses less than 30%) and within 0.25m/s of metabolic treadmill trial speeds were averaged for walking and running in further analysis. From these data, complete walking trials were available for twenty-five individuals and running trials were available for twenty-one individuals. Kinematics data were filtered using a fourth-order zero lag Butterworth filter with a cutoff frequency of 6Hz.

### Magnetic resonance imaging

Full lower body MRIs, scanned isotropically at 1.7mm resolution, were obtained for each subject in the second study group on an Avanto 1.5T scanner at the Center for Clinical Imaging Research, Washington University in St. Louis. Pelvic dimensions, muscle moment arms and architecture of 16 muscles of the lower limb were measured using Analyze 10.0 software, Biomedical Imaging Resource, Mayo Clinic (S1 Text). Three-dimensional coordinate data for the hip, knee and ankle joint centers of rotation taken from subject MRIs were used to create joint landmarks in relation to the filtered kinematics markers on the left anterior superior iliac spine (ASIS), lateral epicondyle and lateral malleolus markers respectively. These landmark points were then used in further calculations of segmental and joint motion. Segment center of mass and moment of inertia were calculated from de Leva (1996)[28] and scaled to subject segment lengths and body mass.

### Hip abductor effective mechanical advantage (EMA)

*EMA* of the hip abductors was measured in two ways, first statically using one-half biacetabular width, measured as the diameter between the right and left hip joint centers viewed from MRI, as a proxy for *R* about the hip (“anatomical *EMA”*). Second, a dynamic measure of *EMA* (“locomotor *EMA*”) was calculated during walking and running force-plate trials using a custom written MATLAB inverse dynamics routine [23,29].

At the hip, the net moment is given by the equation:

$$\sum M_{\text{hip}}\;=\;I_{\text{thigh}}\;\alpha_{\text{thigh}}–M_{\text{thighD}}\;-\;[r_{\text{hip-COM}}\;\times \;g]–[r_{\text{thighD-hip}}\;\times \;F_{\text{thighD}}]$$

where *I*_*thigh*_ is the moment of inertia of the thigh segment about the proximal end resolved using the parallel axis theorem [23], *α*_*thigh*_ is the angular acceleration of the thigh, *M*_*thighD*_ is the moment acting at the distal segment of the thigh, *r*_*hip-COM*_ is the positional vector of the hip relative to the thigh center of mass, g is gravity, *r*_*thighD-hip*_ is the positional vector of the distal thigh segment relative to the hip, and *F*_*thighD*_ is the force vector acting on the distal segment of the thigh. All vector multiplications are cross products. The external moment arm, *R*, acting about the hip was then calculated as:

$$R\;=\;\frac{M_{\text{hip}}\;-\;I_{\text{thigh}}\;\alpha_{\text{thigh}}\;+\;M_{\text{thighD}}\;+\;[r_{\text{hip-COM}}\;\times \;g]}{F_{\text{thighD}}}$$

Values of *R* at midstance of the foot-ground contact period were used for calculations of locomotor *EMA*. For both static “anatomical *EMA*” and dynamic measures of *EMA* during locomotion, the hip abductor moment arm was determined directly from MRI (S1 Text).

### Locomotor cost

Locomotor cost was calculated as the net volume of oxygen consumed during exercise above resting VO_2_ via open flow respirometry (PA-10 Oxygen Analyzer, Sable Systems International) [30,31] on a stationary treadmill at 1.5m/s walking speed (mean±1SD Froude = 0.25±0.04) and 2.5m/s running speed (mean±1SD Froude = 0.69±0.08) for all subjects (where Froude = speed^2^/hip height × gravity). Differences in cost between men and women used the combined metabolic sample from experiments one and two, making net cost data available for a total of forty-one men and women (male n = 21, female n = 20). There were no significant differences in running cost between the two studies (*P* = 0.734), and while average walking costs were slightly higher in the study two group (2.24 J kg^-1^ m^-1^ vs. 2.03 J kg^-1^ m^-1^ for study one, *P* = 0.02) both group values are within average reported cost measures for previous analyses of the metabolic cost of walking [32].

### Hip abductor active muscle volume and cost

Because locomotor cost is closely tied to the amount of muscle volume that must be activated in the stance limb during ground contact [33–35], the contribution of hip abductor force production to the metabolic demand of walking and running was estimated as follows. First, agonist muscle force for the hip abductors and each extensor muscle group of the lower-limb (Table A in S1 Text) was measured as the net moment about the joint determined from inverse dynamics (as above for the hip abductors) divided by the composite muscle moment arm for that group, accounting for the activation of biarticulate muscles at the knee and hip [29]. Then, active muscle volume of each muscle group during stance phase was determined by the equation:

$$V_{muscle}\;=\;L_{fasc}\;\times \;\frac{F_{m}}{\sigma }$$

where *L*_*fasc*_ is the composite muscle fascicle length (S1 Text, Table B in S1 Text) for the muscle group, *F*_*m*_ is muscle force generated by the muscle group to oppose external moments about the joint and σ is a constant of muscle stress (20 N/cm^2^ [29]). A limitation of this method is that calculating active muscle volume assumes uniform specific tension across muscles, which is the case only for isometric contractions [36] and thus not representative of all muscle contractions during gait. Also, we cannot control for variation in muscle fiber type activation (slow vs. fast muscle fibers), which differ in metabolic demand, across gaits. However, small between subject variations in either of these components are unlikely to alter the results presented here.

To determine the metabolic cost of activating a given amount of muscle volume, net locomotor cost (J m^-1^) was regressed on summed lower limb active muscle volume (S1 Fig.), and the slope of this regression line was used to calculate the expected mass-specific cost (J kg^-1^ m^-1^) of hip abductor force production for each subject during walking and running. Hip abductor percentage of total locomotor cost was calculated as the mass-specific hip abductor cost divided by observed locomotor cost for each individual. Because this approach ignores the cost of leg-swing, which may account for between 10–30% of total locomotor cost [37], our estimates for hip abductor contribution to the overall cost of walking and running are likely slight overestimates. However, ignoring swing cost should not affect male-female comparisons of hip abductor force production.

### Statistics

Student’s two-tailed t-tests were used to assess differences in means between males and females using the Holm-Bonferroni correction for family-wise error to assess significance [38,39]. All anthropometric variables were considered a single family, while biomechanical and cost variables were treated as a separate family of analyses. All regressions are linear least squares. Individual subject data for all anthropometric, biomechanical and metabolic measurements are available in the Supporting Information (Tables C-E in S1 Text).

## Results

### Hip abductor mechanics

The standard static biomechanical model of hip abductor force production predicts that hip abductor *EMA* is lower in women due to greater biacetabular width, thus increasing locomotor cost. Women did have significantly lower anatomical *EMA* than men (*P* = 0.006, Table 1), but locomotor *EMA* measured dynamically, although somewhat lower in women, was not significantly different after a Holm-Bonferroni correction (walk, *P* = 0.05; run, *P* = 0.01, Table 2). Contrary to the expectations of the obstetrical dilemma, differences in anatomical *EMA* did not derive from pelvic width. The most relevant biomechanical measure of biacetabular width, measured as the diameter between the centers of the femoral heads, was not significantly different between men and women (*P* = 0.16) despite women having greater bispinous (*P* < 0.001) and mediolateral outlet (*P* = 0.002) diameters, measures that are more directly relevant to obstetric function than biacetabular width [10,40] (Table 1). The lack of difference in biacetabular width resulted from femoral head diameters that were 10% larger in males than females (*P* < 0.001), which translates the joint’s center of rotation laterally relative to the body midline. Importantly, biacetabular width was not a strong predictor of dynamic measures of *R* during walking or running (walk, R^2^ = 0.05, *P* = 0.28; R^2^ = 0.06, *P* = 0.28; S2 Fig.;[18]), and dynamic measurements of *R* did not differ significantly between men and women at either a walk (*P* = 0.24) or a run (*P* = 0.05, Table 2) after correction for multiple comparisons.

**Table 1:** Summary statistics for anthropometric measurements.

	female	male	total	*p*-value
body mass (kg)	60.9±7.7	69.1±8.2	64.9±8.7	**0.01**
height (m)	1.64±0.03	1.77±0.06	1.71±0.08	**< 0.0001**
abductor r (cm)	5.2±0.4	5.9±0.8	5.6±0.7	**0.01**
femoral neck (cm)	5.8±0.2	6.5±0.6	6.1±0.5	**0.004**
bispinous breadth (cm)	11.3±0.8	9.6±0.8	10.5±1.1	**< 0.0001**
ML outlet (cm)	12.8±1.3	11.3±0.8	12.1±1.4	**0.002**
biacetabular width (cm)	17.6±0.6	17.2±0.5	17.4±0.6	0.16
femoral head diameter (cm)	4.1±0.1	4.5±0.2	4.3±0.3	**< 0.0001**
anatomical EMA	0.59±0.08	0.69±0.09	0.64	**0.006**

Mean ± standard deviation. **Bold** values indicate statistical significance at the Holm-Bonferroni alpha level [36, 37].

**Table 2:** Hip abductor mechanics and cost comparisons in males and females.

	walk			run		
	female	male	*p-*value	female	male	*p-*value
*EMA* (*r/R*)	0.98±0.17	1.49±0.88	0.05	0.83±0.26	1.35±0.56	0.01
*R* (cm)	5.5±0.9	4.8±1.8	0.23	6.5±1.4	5.1±1.6	0.05
hip abductor cost (J kg^-1^ m^-1^)	0.16±0.03	0.12±0.03	**0.006**	0.39±0.07	0.29±0.07	**0.006**
locomotor cost (J kg^-1^ m-^1^)	2.19±0.32	2.14±0.29	0.63	3.49±0.43	3.48±0.48	0.94

Effective mechanical advantage (*EMA*), *R*, and hip abductor cost estimates determined at a walk (female n = 13, male n = 12) and a run (female n = 10, male n = 11) for subjects who participated in kinematics, metabolic and MRI trials. Net locomotor cost determined for a combined sample of subjects who participated in walking (female n = 19, male n = 20) and running (female n = 13, male n = 14) metabolic trials. Mean ± standard deviation. **Bold** values indicate statistical significance at the Holm-Bonferroni alpha level [38, 39].

The significantly lower anatomical *EMA* observed in women, and the slight but non-significant differences between women and men in locomotor *EMA*, are not the result of differences in pelvic width but instead derive primarily from shorter hip abductor moment arms, *r*, in women (*P* = 0.01, Table 1). Greater femoral neck length and biiliac breadth both influence the length of *r* by moving the hip abductor origin and attachment sites farther from the joint center of rotation [12,19]. Abductor moment arm length correlated significantly with femoral neck length (R^2^ = 0.62, *P* < 0.001; S3a Fig.), which itself correlated with total femoral length (R^2^ = 0.49, *P* < 0.001) and is clearly influenced by overall body size [19]. Additionally, biiliac width was also significantly correlated with *r* (R^2^ = 0.51, *P* < 0.001; S3b Fig.). The strong relationship between biiliac width and femoral neck length with *r* demonstrate that both body size and shape can influence hip abductor function. However, the poor relationship between biacetabular width and *R* (even though men and women were clearly dimorphic in obstetrically relevant aspects of pelvic shape), the marked differences between anatomical *EMA* and locomotor *EMA*, and the significant between subject variation in locomotor *EMA* itself, indicates hip abductor mechanics are more complex than the standard static model suggests.

### Locomotor cost, hip abductor force production and pelvic width

**Fig 2:**
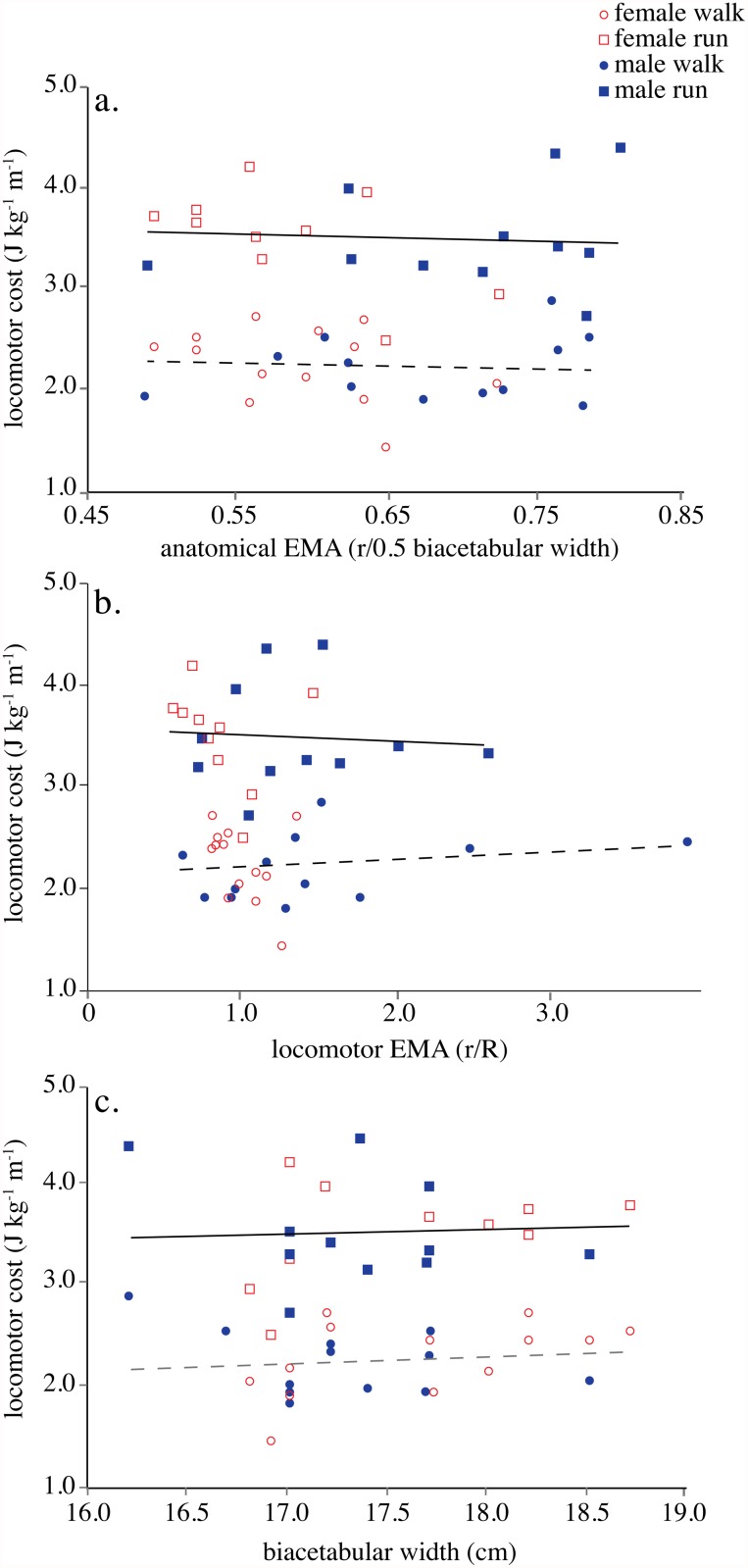
Hip abductor *EMA* measured anatomically and dynamically versus locomotor cost. a. Locomotor cost versus anatomical *EMA* derived from the static model at a walk (y = -0.29x + 2.43, R^2^ = 0.006, *P* = 0.71) and a run (y = -0.35x + 3.73, R^2^ = 0.005, *P* = 0.75). b. Locomotor cost versus locomotor *EMA* determined dynamically at a walk (y = -0.07x + 2.15, R^2^ = 0.02, *P* = 0.49) and a run (y = -0.06x + 3.57, R^2^ = 0.004, *P* = 0.77). c. Biacetabular width versus locomotor cost at a walk (y = 6.85x + 1.04, R^2^ = 0.02, *P* = 0.54) and a run (y = 4.52x + 2.71, R^2^ = 0.003, *P* = 0.81). Lines indicate ordinary LSR (walk, n = 25; run, n = 21).

Contrary to the expectations of the obstetrical dilemma, locomotor cost was not predicted by either static anatomical or dynamic measures of hip abductor *EMA* during walking (anatomical *EMA*, R^2^ = 0.006, *P* = 0.71; locomotor *EMA*, R^2^ = 0.02, *P* = 0.49) or running (anatomical *EMA*, R^2^ = 0.005, *P* = 0.75; locomotor *EMA*, R^2^ = 0.004, *P* = 0.77; Fig. 2a and 2b). Absolute biacetabular width was also a poor predictor of locomotor cost during walking (R^2^ = 0.017, *P* = 0.53) and at a run (R^2^ = 0.003, *P* = 0.81; Fig. 2c). Because females in our sample were significantly shorter than males but had equivalent biacetabular diameters, we tested whether leg length influenced our findings by regressing cost on biacetabular width relative to leg length. There was no significant relationship between relative biacetabular width and cost at either gait (walk, R^2^ = 0.018, *P* = 0.52; run, R^2^ = 0.002, *P* = 0.84). These results were also independent of body mass, which was not significantly correlated with mass-specific locomotor cost (walk, R^2^ = 0.02, *P* = 0.49; run, R^2^ = 0.07, *P* = 0.23).

**Fig 3:**
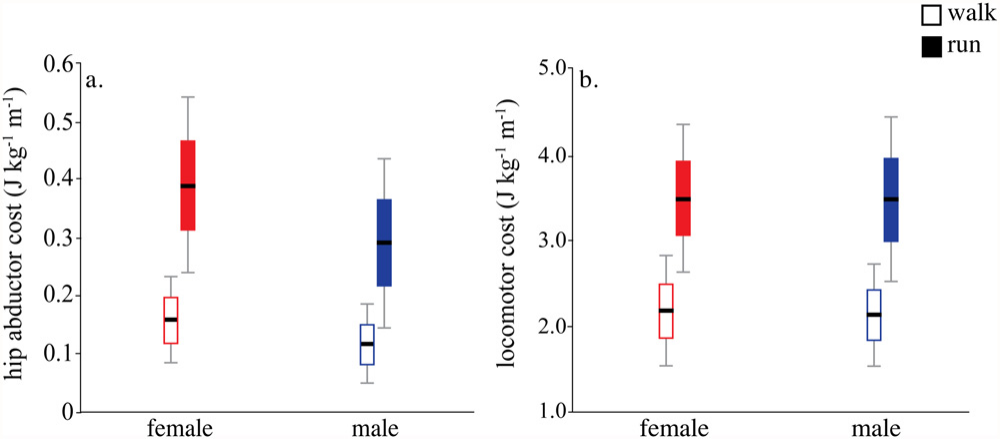
Metabolic cost of hip abductor activation and net locomotor cost in men and women. a. Hip abductor mass-specific cost is higher in women at both a walk (*P* = 0.006) and a run (*P* = 0.006) due to lower EMA from shorter average abductor moment arm length (see text, Table 2) b. Net mass-specific locomotor cost does not differ between men and women at a walk or a run. Black bar indicates mean, box indicates mean±1SD and whiskers are mean±2SD.

Hip abductor cost estimated from the active muscle volume cost regression was 35% higher in women than men during both walking (*P* = 0.006) and running (*P* = 0.006) due to lower hip abductor *EMA* in women (Fig. 3a, Table 2). However, because the hip abductors accounted for a relatively small proportion of the total cost of walking (6.3±1.8%) and running (9.7±2.3%) (also see [41,42]), net mass-specific locomotor economy did not differ significantly between men and women (walk, *P* = 0.63; run, *P* = 0.94; Fig. 3b, Table 2). Therefore, the differences between men and women in hip abductor *EMA* and cost attributed to smaller hip abductor *r* were not great enough to influence overall locomotor economy.

## Discussion

Our results suggest that the static biomechanical model that underlies the obstetrical dilemma trade-off hypothesis that a wider pelvis is required to permit the birth of large-brained infants but a narrow pelvis increases locomotor efficiency does not accurately represent dynamic hip abductor mechanics during locomotion. Additionally, biacetabular width is not correlated with locomotor cost, and hip abductor force production appears to only minimally influence total metabolic expenditure during walking and running. As previously shown [25–27] there is no significant difference in locomotor efficiency between men and women. These data indicate that while pelvic shape in female humans was selected to accommodate the birth of large-brained neonates, locomotor efficiency has not been compromised by obstetric function. Instead, skeletal measures such as femoral neck length and biiliac width that are dependent on body size and shape are more important for determining hip abductor *EMA* by influencing the length of *r*. The variability of *R* measured dynamically suggests that mediolateral *GRF*, lower limb kinematics and body center of mass displacement interact in complex ways to determine the magnitude of hip abductor force production. Two subjects in particular (subjects 14 and 39) stand out with exceptionally high locomotor *EMA* due to a combination of long *r* and very short *R* (Fig. 2b, Tables C and D in S1 Text). Their values of *R* were similar across multiple walking and running trials indicating a consistent locomotor pattern. However, no clear kinematic or kinetic cause for their uniquely small values of *R* were apparent.

One limitation of our study is that all participants walked and ran at the same speed for locomotor cost trials. These speeds were chosen to maximize the comparable sample between force and metabolic trials because many participants traveled at relatively slow speeds on the force track-way despite verbal instructions to travel at “slow,” “preferred,” and “fast” speeds for different trials. Additionally, because the biomechanical methods of this study necessarily require locomotor testing to take place on level, indoor surfaces (i.e. force-plate trackway and treadmill), the estimates for hip abductor cost may underestimate the cost of activating these muscles when walking and running on more naturalistic, uneven terrain, or when carrying a burden. This analysis also does not account for variation in axial kinematics and muscle activity in the trunk during locomotion that could potentially be related to pelvic width. However, our analysis of cost versus biacetabular width would likely have indicated if pelvic width was directly influencing axial muscle function in a manner that was metabolically expensive. Furthermore, the variability evident in dynamic measures of *EMA*, during even constrained locomotor conditions highlights the importance of looking beyond simple measures of pelvic width for understanding mediolateral loading at the hip. Future research is needed to better tease apart the kinetic and kinematic factors that influence hip dynamics.

The results reported here present a broader conundrum for understanding the obstetrical dilemma: if wider pelves do not increase locomotor cost, why hasn’t selection favored even wider female pelves to reduce the risk of birth complications from cephalopelvic disproportion (CPD)? Several hypotheses may explain this problem. One possibility is that selection has favored narrower pelves for other aspects of locomotor performance such as reducing injury or increasing speed. While the risk of certain knee injuries is 4–6 times greater in female athletes than male athletes competing in high-risk sports [43], static knee valgus angle, influenced by greater relative biacetabular width, is not correlated with dynamic loading of the knee or injury occurrence [43,44]. This suggests that higher injury rates among women result from other factors such as less neuromuscular control or muscle strength [44]. Speed is also an unlikely factor restricting pelvic width because maximum speed is primarily determined by the ability to increase ground contact forces [45]. Therefore slower running speeds in women [46] are likely driven by relatively less muscle mass, relatively more adipose tissue, and lower anaerobic and aerobic capacities in women [47].

An alternative hypothesis is that pelvic width is constrained by thermoregulatory demands on body breadth [1,2,48]. The biiliac breadth of the pelvis varies ecogeographically and is smallest in low latitude populations where minimizing heat production through a decrease in body mass is thought to be advantageous [2]. While biiliac breadth is correlated with mediolateral dimensions of the birth canal at population level comparisons of geographically diverse groups [49], the obstetric capacity of the birth canal appears to be maintained in smaller bodied populations by increases in the anteroposterior diameters of the lower pelvis [49,50]. Although it is not clear how strongly the correlations between biiliac breadth and mediolateral midplane and outlet dimensions are at the individual level, these broader comparisons suggest that selection on the pelvis for thermoregulation and birth are not necessarily antagonistic [49,51].

A third hypothesis is that current rates of CPD reflect two divergent effects of high-energy, low-nutrient agricultural diets [52]. First, decreases in stature and increases in disease are clearly associated with the agricultural transition across populations [53]. This type of malnutrition, as well as Vitamin D insufficiency due to lack of sunlight exposure, can significantly reduce pelvic growth during development and has been linked to maternal mortality due to obstructed labor in both contemporary [54–57] and historical populations [52,58]. Second, high to excessive levels of maternal energy during pregnancy, which used to be rare, have the potential to increase fetal size beyond the capacity of the mother’s birth canal. Maternal obesity (defined as BMI > 40) increases the risk of delivering a macrosomic infant (birth weight ≥ 4000g) nearly 3-fold [59,60]. Such increases in fetal size have been shown to increase the rate of CPD and shoulder dystocia; 6% and 11% respectively compared to 2.1% and 2.4% for demographically matched deliveries where infant size was below 4000g [61]. This hypothesis, however, is difficult to test. Obtaining data on birth outcomes in hunter-gatherer societies, where nutritional status throughout maternal growth and pregnancy is likely to more accurately reflect the energy environment in which most of human evolution occurred, is necessary to help clarify how representative current rates of obstructed labor are for interpreting selection on the female pelvis.

While the obstetrical dilemma has been the primary model for explaining why human childbirth is so difficult, the absence of evidence for increased locomotor cost with greater pelvic width suggests that this aspect of the model needs to be reconsidered. Although there is undoubtedly a tight fit between the maternal pelvis and fetal head, our analysis shows that factors other than selection for locomotor economy must be necessary to explain the variable occurrence of CPD in modern human populations. Additional research is needed to understand current rates of CPD in the context of variations in maternal nutrition and energy availability across populations, and to understand the ecological and evolutionary pressures affecting human pelvic morphology.

## Supporting Information

S1 Text Muscle moment arm, cross-sectional area and fascicle length. Table A in S1 Text. Muscle groups included for determination of total lower limb active muscle volume during walking and running.
Table B in S1 Text. Muscle fiber lengths and ratios from cadaveric specimens.
Table C in S1 Text. Subject anthropometrics.
Table D in S1 Text. *EMA* and *R* measured during locomotion.
Table E in S1 Text. Net locomotor cost for all subjects.
(DOCX)

S1 Fig Lower limb active muscle volume versus locomotor cost. The metabolic demand of the hip abductors was estimated using the slope of the regression relating net body locomotor cost to lower limb active muscle volume (Table A) required to travel one meter at a walk and a run. Line indicates mixed model regression controlling for repeated measures (slope = 0.024, *P* < 0.001; y-intercept = 79.28, *P* < 0.001).
(TIF)

S2 Fig Biacetabular width versus *R*. Biacetabular width, defined as the distance between the centers of the femoral heads measured on MRI, is not significantly correlated with *R* at mid-stance during walking (y = 0.51x –3.7, R^2^ = 0.05, *P* = 0.28, n = 25) or running trials (y = 0.65x –5.7, R^2^ = 0.06, *P* = 0.28, n = 21). This result is consistent with previous analyses [18]. Lines indicate OLS regressions.
(TIF)

S3 Fig Hip abductor *r* versus femoral neck length and biiliac width. Hip abductor moment arm length, *r*, is significantly correlated with femoral neck length (y = 1.0x –0.62, R^2^ = 0.62, *P* < 0.0001) b. and biiliac width (y = 0.32x –2.9, R^2^ = 0.51, P < 0.001) in males (n = 13) and females (n = 13). Lines indicate OLS regressions.
(TIF)
