# Supplementary material for: A Wider Pelvis Does Not Increase Locomotor Cost in Humans, with Implications for the Evolution of Childbirth
Source: PLoS One. 2015 Mar 11;10(3):e0118903. doi: 10.1371/journal.pone.0118903 (PMC4356512; doi:10.1371/journal.pone.0118903)
Supplement: S1 Text — Table A in S1 Text. Muscle groups included for determination of total lower limb active muscle volume during walking and running. Table B in S1 Text. Muscle fiber lengths and ratios from cadaveric specimens. Table C in S1 Text. Subject anthropometrics. Table D in S1 Text. EMA and R measured during locomotion. Table E in S1 Text. Net locomotor cost for all subjects. (DOCX) [file pone.0118903.s001.docx]

**S1 Text. Muscle moment arm, cross-sectional area and fascicle length**

Muscle moment arms and cross-sectional areas of the muscles comprising the hip abductor, hip and knee extensor and ankle plantarflexor muscle groups (Table A) were measured from subject MRI using Analyze 10.0 software. Individual muscle moment arms were calculated as the perpendicular distance from the joint center of rotation (hip, knee or ankle) to the muscle line of action defined by points perpendicular to the muscle’s origin and insertion and passing through the center of the muscle body. Each muscle’s moment arm was measured in the plane of primary action during walking and running (i.e. coronal for hip abductors and sagittal for extensor muscle groups). Maximum cross-sectional area of each muscle was measured by manually defining the borders of the muscle in the transverse plane until the largest planer area had been found. For each muscle group, the composite moment arm, *r*, was calculated as:

*r* = *r_1_* × *A_1_*/*A_tot_* + *r_2_* × *A_2_*/*A_tot_* + …*r_i_* × *A_i_*/*A_tot_*

where *r_1…i_* is each individual muscle’s moment arm, *A_1…i_* is each muscle’s maximum cross sectional area and *A_tot_* = *A_1_* + *A_2_* +…*A_i_* [29].

Individual muscle fascicle lengths for each of the 16 muscles included in the analysis were estimated based on a ratio of fascicle length to total muscle-tendon length measured on 4 cadaveric specimens (male = 2, female = 2, age = 81.7±14.5y). Average fascicle lengths and fascicle to muscle tendon length ratios measured from cadavers are given in Table B. Subject specific values were normalized based on individual muscle lengths obtained from MRI. Muscle group composite fascicle length for each subject was calculated in the same manner as muscle moment arms.

| **Table A. Muscle groups included for determination of total lower limb active muscle volume during walking and running.** | |
| --- | --- |
| **Group** | **Muscle** |
| Hip abductors | Gluteus medius, gluteus minimus, tensor fasciae latae |
| Hip extensors | Gluteus maximus, semitendinosus, semimembranosus, biceps femoris (long and short heads) |
| Knee extensors | Rectus femoris, vastus lateralis, vastus intermedius and vastus medialis |
| Ankle plantarflexors | Gastrocnemius (medialis and lateralis), soleus, tibialis posterior, flexor hallucis longus, flexor digitorum longus |

| Table B. Muscle fiber lengths and ratios from cadaveric specimens. | | |
| --- | --- | --- |
| Muscle | **Fiber length (cm)** | **Fiber length/muscle-tendon length** |
| Gluteus maximus | 13.47 | 0.498 |
| Gluteus medius | 6.53 | 0.331 |
| Gluteus minimus | 3.60 | 0.271 |
| Tensor fasciae latae | 12.75 | 0.255 |
| Semitendinosus | 8.84 | 0.189 |
| Semimembranosus | 6.35 | 0.151 |
| Biceps femoris (long) | 8.40 | 0.188 |
| Biceps femoris (short) | 11.28 | 0.396 |
| Rectus femoris | 5.52 | 0.132 |
| Vastus lateralis | 8.31 | 0.224 |
| Vastus intermedius | 8.10 | 0.244 |
| Vastus medialis | 7.93 | 0.227 |
| Gastrocnemius (medialis) | 4.35 | 0.098 |
| Gastrocnemius (lateralis) | 5.35 | 0.123 |
| Soleus | 4.05 | 0.104 |
| Tibialis posterior | 4.03 | 0.108 |
| Flexor hallucis longus | 4.80 | 0.160 |
| Flexor digitorum longus | 3.81 | 0.117 |

| **Table C. Subject anthropometrics.** | | | | | | | |
| --- | --- | --- | --- | --- | --- | --- | --- |
| **Subject** | **Sex** | **Height (m)** | **Body mass (kg)** | **Biacetabular width (cm)** | **Biiliac width (cm)** | **Femoral neck length (cm)** | **Hip abductor r (cm)** |
| 14 | M | 1.85 | 73.6 | 17.7 | 28.4 | 7.9 | 7.0 |
| 15 | M | 1.76 | 70.1 | 17.0 | 28.4 | 6.3 | 6.7 |
| 17 | M | 1.72 | 69.8 | 17.0 | 25.7 | 6.5 | 5.8 |
| 18 | M | 1.77 | 66.1 | 17.0 | 26.2 | 6.4 | 6.2 |
| 19 | F | 1.66 | 47 | 17.7 | 25.3 | 5.5 | 4.6 |
| 20 | F | 1.67 | 78.6 | 16.8 | 28.1 | 5.9 | 6.1 |
| 21 | F | 1.67 | 67.4 | 16.9 | 27.7 | 6.5 | 5.5 |
| 22 | M | 1.70 | 64.7 | 16.7 | 23.1 | 5.7 | 5.1 |
| 23 | M | 1.67 | 58.8 | 17.7 | 22.7 | 5.9 | 4.3 |
| 24 | F | 1.65 | 60.8 | 18.0 | 26.1 | 6.0 | 5.4 |
| 25 | F | 1.58 | 57.8 | 17.0 | 24.3 | 5.6 | 4.8 |
| 26 | M | 1.8 | 73.3 | 18.5 | 27.2 | 6.7 | 5.8 |
| 27 | F | 1.66 | 63.5 | 17.7 | 27.5 | 6.0 | 5.6 |
| 28 | M | 1.80 | 64.6 | 17.4 | 28.1 | 6.3 | 6.2 |
| 29 | M | 1.75 | 59.5 | 17.2 | 25.2 | 5.6 | 5.0 |
| 30 | M | 1.86 | 71.1 | 17.7 | 27.5 | 6.1 | 5.6 |
| 31 | F | 1.66 | 58.1 | 18.5 | 26.9 | 6.1 | 5.9 |
| 32 | F | 1.71 | 58.1 | 18.2 | 25.0 | 5.5 | 4.5 |
| 33 | M | 1.72 | 64.5 | 16.2 | 26.4 | 7.2 | 6.2 |
| 34 | F | 1.65 | 61.2 | 18.2 | 29.1 | 6.0 | 5.1 |
| 35 | F | 1.60 | 63.2 | 17.2 | 26.9 | 6.0 | 5.2 |
| 36 | F | 1.62 | 54.9 | 17.0 | 25.7 | 5.8 | 4.8 |
| 37 | F | 1.67 | 64.5 | 18.7 | 25.5 | 6.0 | 4.9 |
| 39 | M | 1.80 | 91.4 | 17.2 | 27.4 | 6.4 | 6.6 |
| 40 | M | 1.85 | 70.3 | 17.3 | 29.1 | 7.3 | 7.0 |
| 41 | F | 1.61 | 55.9 | 17.2 | 27.5 | 5.7 | 5.5 |
| Measurements for 26 subjects who participated in kinematics, metabolic and MRI trials. | | | | | | | |

| **Table D. *EMA* and *R* measured during locomotion.** | | | | | |
| --- | --- | --- | --- | --- | --- |
| **Subject** | **Sex** | ***R* walk (cm)** | ***EMA* walk** | ***R* run (cm)** | ***EMA* run** |
| 14 | M | 2.0 | 3.82 | 2.7 | 2.59 |
| 15 | M | 5.2 | 1.28 | 6.5 | 1.03 |
| 17 | M | 3.4 | 1.76 | 3.5 | 1.62 |
| 18 | M | 6.7 | 0.92 | 8.3 | 0.75 |
| 19 | F | 5.6 | 0.82 | 6.6 | 0.70 |
| 20 | F | 6.5 | 0.94 | 6.0 | 1.03 |
| 21 | F | 4.7 | 1.24 | 5.6 | 0.987 |
| 22 | M | 3.9 | 1.32 | * | * |
| 23 | M | 5.8 | 0.75 | 6.2 | 0.7 |
| 24 | F | 4.7 | 1.14 | 6.5 | 0.84 |
| 25 | F | 4.5 | 1.08 | 5.8 | 0.83 |
| 26 | M | 4.2 | 1.38 | 4.2 | 1.4 |
| 27 | F | 6.2 | 0.91 | * | * |
| 28 | M | 6.8 | 0.92 | 5.4 | 1.16 |
| 29 | M | 8.3 | 0.60 | * | * |
| 30 | M | 4.8 | 1.15 | 5.9 | 0.94 |
| 31 | F | 6.8 | 0.87 | * | * |
| 32 | F | 5.6 | 0.81 | 7.5 | 0.60 |
| 33 | M | 4.1 | 1.51 | 5.4 | 1.14 |
| 34 | F | 6.6 | 0.77 | 6.7 | 0.76 |
| 35 | F | 5.8 | 0.90 | * | * |
| 36 | F | 4.5 | 1.05 | 7.3 | 0.65 |
| 37 | F | 6.1 | 0.81 | 9.2 | 0.53 |
| 39 | M | 2.7 | 2.46 | 3.3 | 2.0 |
| 40 | M | * | * | 4.7 | 1.49 |
| 41 | F | 4.2 | 1.33 | 3.8 | 1.43 |
| *EMA* and *R* measured at mid-stance from kinematics trials using an inverse dynamics approach. * indicates missing data. | | | | | |
|  | | | | | |

| **Table E. Net locomotor cost for all subjects.** | | | | |  |
| --- | --- | --- | --- | --- | --- |
| **Subject** | **Sex** | **Body Mass (Kg)** | **Walk (J kg^-1^ m^-1^)** | **Run (J kg^-1^ m^-1^)** | **Study Location** |
| 14 | M | 73.6 | 2.518 | 3.349 | WU |
| 15 | M | 70.12 | 1.823 | 2.724 | WU |
| 17 | M | 69.8 | 1.923 | 3.250 | WU |
| 18 | M | 66.1 | 2.011 | 3.484 | WU |
| 19 | F | 47 | 2.465 | 3.660 | WU |
| 20 | F | 78.65 | 2.062 | 2.925 | WU |
| 21 | F | 67.4 | 1.456 | 2.480 | WU |
| 22 | M | 64.7 | 2.519 | * | WU |
| 23 | M | 58.78 | 1.920 | 3.217 | WU |
| 24 | F | 60.87 | 2.152 | 3.559 | WU |
| 25 | F | 57.87 | 2.160 | 3.281 | WU |
| 26 | M | 73.39 | 2.040 | 3.280 | WU |
| 27 | F | 63.5 | 1.928 | * | WU |
| 28 | M | 64.59 | 1.944 | 3.136 | WU |
| 29 | M | 59.51 | 2.324 | * | WU |
| 30 | M | 71.12 | 2.272 | 3.961 | WU |
| 31 | F | 58.1 | 2.430 | * | WU |
| 32 | F | 58.6 | 2.425 | 3.719 | WU |
| 33 | M | 64.5 | 2.875 | 4.342 | WU |
| 34 | F | 61.23 | 2.693 | 3.495 | WU |
| 35 | F | 63.23 | 2.539 | * | WU |
| 36 | F | 54.97 | 1.905 | 4.168 | WU |
| 37 | F | 64.5 | 2.526 | 3.786 | WU |
| 39 | M | 91.44 | 2.396 | 3.404 | WU |
| 40 | M | 70.3 | * | 4.410 | WU |
| 41 | F | 55.88 | 2.692 | 3.919 | WU |
| 42 | M | 74.4 | 2.115 | 3.278 | WU |
| 43 | M | 76.8 | 2.455 | 3.823 | WU |
| 44 | M | 65.1 | 2.051 | 3.127 | WU |
| 45 | F | 52.8 | 2.036 | * | H |
| 46 | F | 54.3 | 2.251 | 3.702 | H |
| 47 | F | 58 | 1.919 | * | H |
| 48 | F | 73 | 1.887 | 3.373 | H |
| 49 | M | 78.3 | 2.204 | * | H |
| 50 | M | 71.2 | 1.702 | * | H |
| 51 | M | 69.4 | 1.958 | * | H |
| 52 | M | 78.1 | 2.025 | * | H |
| 53 | F | 53.7 | 1.954 | * | H |
| 54 | F | 63.1 | 2.128 | * | H |
| 55 | M | 88.2 | 1.768 | * | H |
| 56 | F | 61.3 | * | 3.396 | H |
| * indicates missing data. Study locations are Washington University in St. Louis (WU) and Harvard University (H). | | | | |  |
