# Supplementary figures and images for: A Wider Pelvis Does Not Increase Locomotor Cost in Humans, with Implications for the Evolution of Childbirth
Source: PLoS One. 2015 Mar 11;10(3):e0118903. doi: 10.1371/journal.pone.0118903 (PMC4356512; doi:10.1371/journal.pone.0118903)

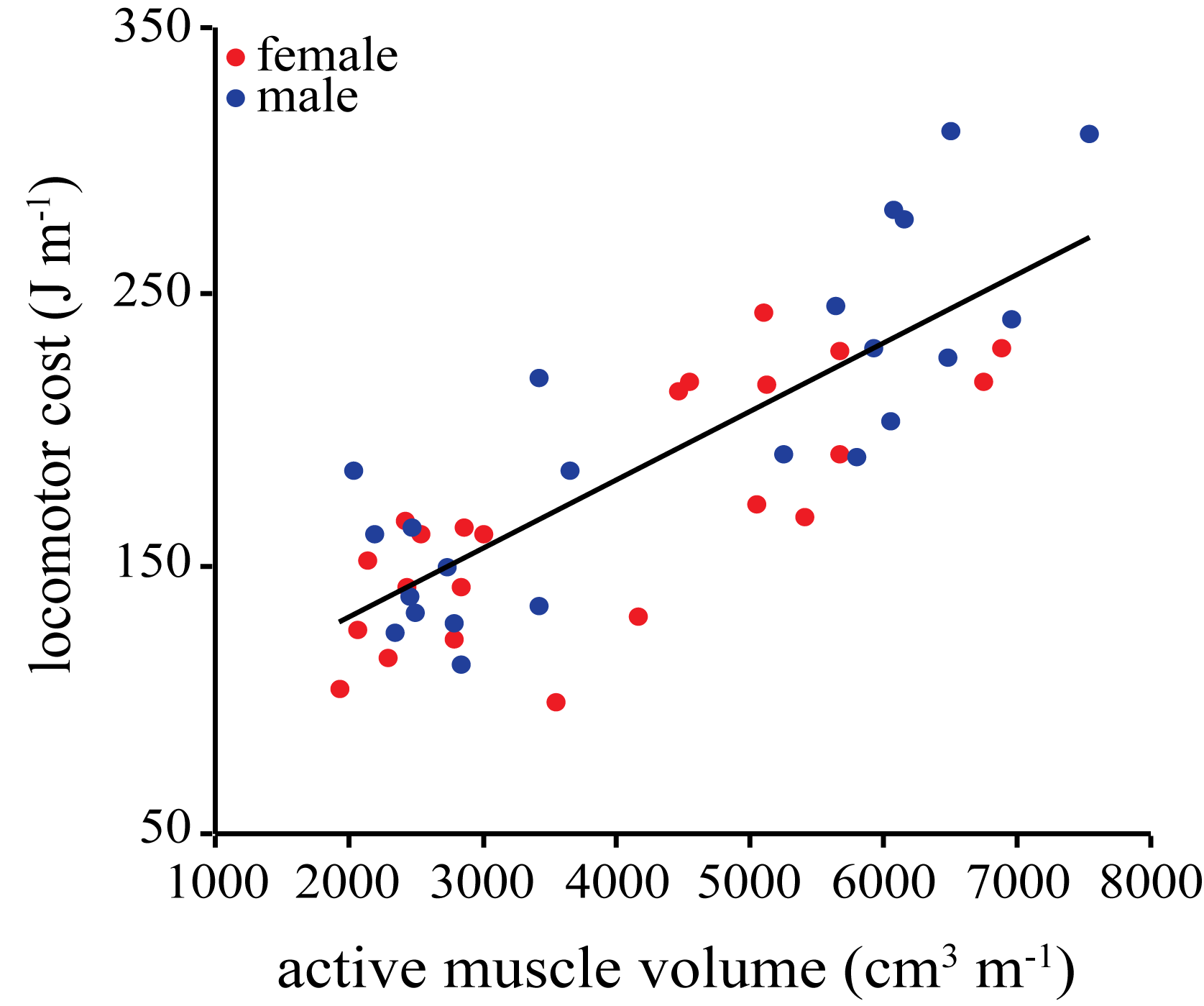

Supplement: S1 Fig — The metabolic demand of the hip abductors was estimated using the slope of the regression relating net body locomotor cost to lower limb active muscle volume (Table A) required to travel one meter at a walk and a run. Line indicates mixed model regression controlling for repeated measures (slope = 0.024, P < 0.001; y-intercept = 79.28, P < 0.001). (TIF) [file pone.0118903.s002.tif]

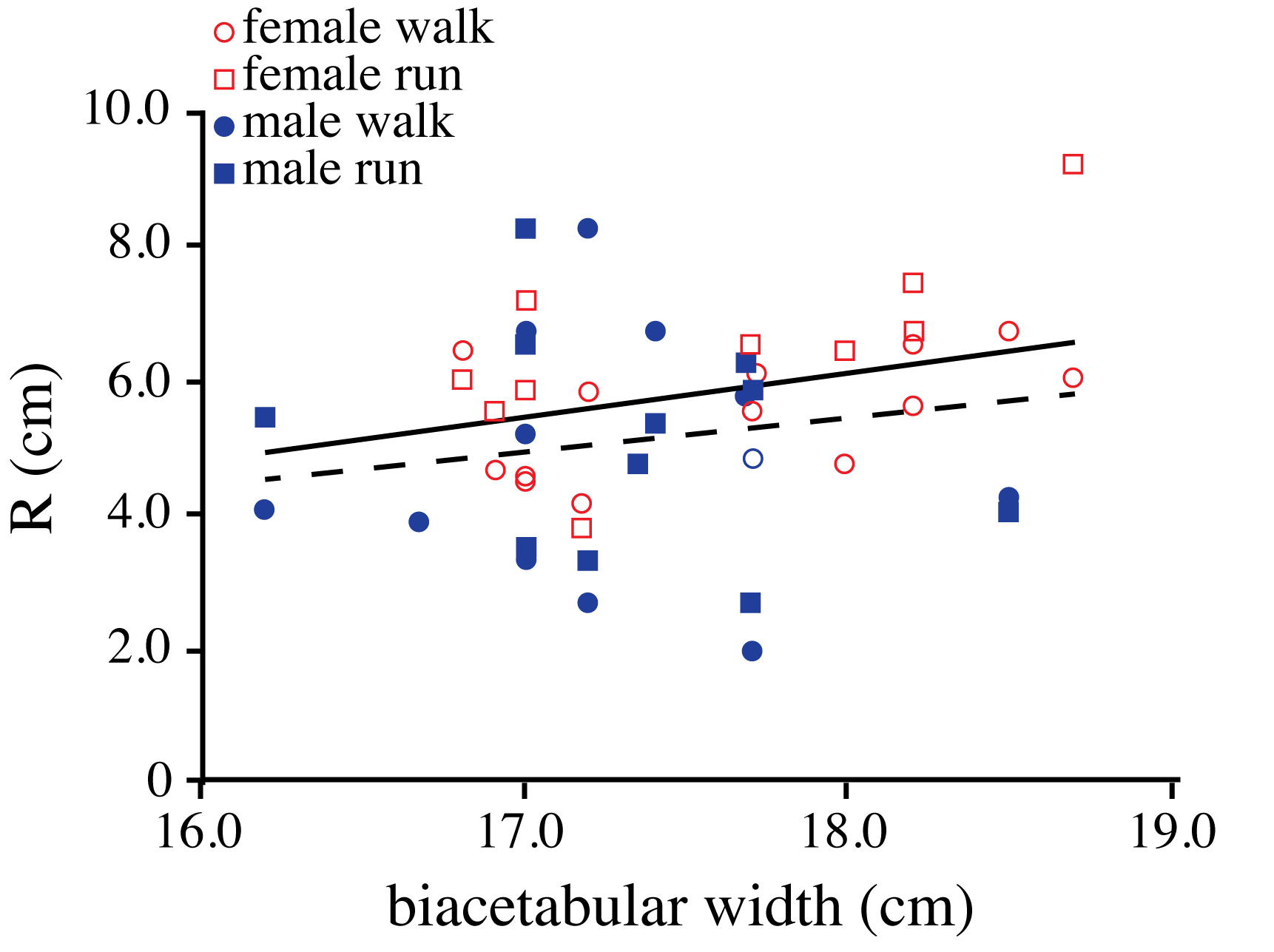

Supplement: S2 Fig — Biacetabular width, defined as the distance between the centers of the femoral heads measured on MRI, is not significantly correlated with R at mid-stance during walking (y = 0.51x –3.7, R2 = 0.05, P = 0.28, n = 25) or running trials (y = 0.65x –5.7, R2 = 0.06, P = 0.28, n = 21). This result is consistent with previous analyses [18]. Lines indicate OLS regressions. (TIF) [file pone.0118903.s003.tif]

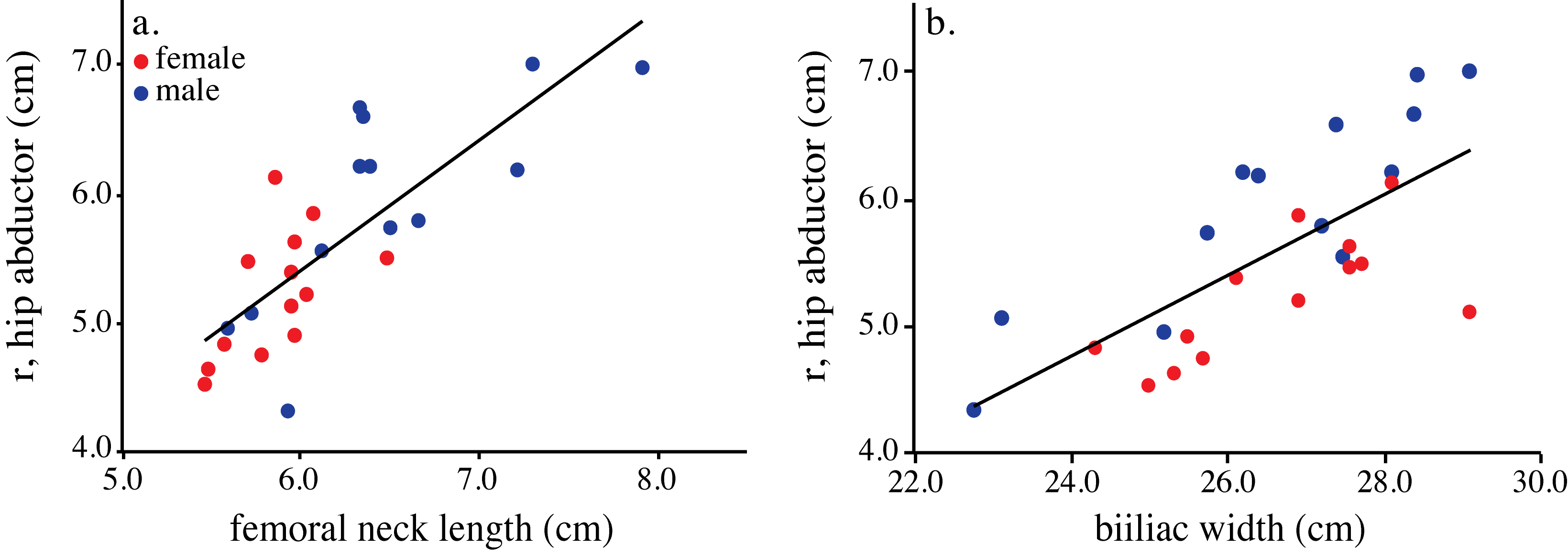

Supplement: S3 Fig — Hip abductor moment arm length, r, is significantly correlated with femoral neck length (y = 1.0x –0.62, R2 = 0.62, P < 0.0001) b. and biiliac width (y = 0.32x –2.9, R2 = 0.51, P < 0.001) in males (n = 13) and females (n = 13). Lines indicate OLS regressions. (TIF) [file pone.0118903.s004.tif]
